# Supplementary material for: Gastrointestinal Microorganisms and Blood Metabolites in Holstein Calves with Different Heat Stress Responses in the Same Hot Environment
Source: Microorganisms. 2025 Mar 31;13(4):801. doi: 10.3390/microorganisms13040801 (PMC12029475; doi:10.3390/microorganisms13040801)
Supplement: Supplementary file 1 [file microorganisms-13-00801-s001.zip › microorganisms-3480876-supplementary.pdf]

Supplemental Table S1. Information on the sequencing depth of 16S rRNA genes in ruminal microbiota

Sample ID	Total read bases (bp)	Clean reads	Mean_length	Min_length	Max_length	Good's-coverage
RH1	27746057	66340	418.240232	204	535	0.998852
RH2	25150535	61088	411.70991	252	449	0.99898
RH3	23290349	56681	410.902225	289	430	0.999298
RH4	19198223	46828	409.973157	262	432	0.999745
RH5	28331078	67756	418.133863	205	540	0.998565
RH6	23084471	55738	414.160375	251	433	0.999745
RH7	29527750	70439	419.196042	222	454	0.998023
RH8	24295569	58246	417.119957	203	470	0.999394
RH9	26243163	63687	412.064676	317	433	0.999745
RH10	25686352	61035	420.846269	252	433	0.999394
RL1	23730086	56983	416.4415	246	440	0.999362
RL2	27429583	66207	414.300346	227	516	0.998661
RL3	22499262	54850	410.196208	400	520	0.999235
RL4	28750491	69060	416.311772	219	433	0.998565
RL5	31529547	75476	417.742686	252	489	0.99662
RL6	20724780	50180	413.008768	335	432	0.999554
RL7	18654120	44730	417.038229	388	432	0.999745
RL8	26428089	62970	419.69333	219	445	0.998884
RL9	32286571	78135	413.215217	231	511	0.998533
RL10	30918834	74715	413.82365	219	436	0.998661
Summation		1241144				
Average	25775245.5	62057.2	415.2059206	259.65	463.15	0.99897805

Supplemental Table S2. Information on the sequencing depth of 16S rRNA genes in intestinal microbiota

Sample ID	Total read bases (bp)	Clean reads	Mean_length	Min_length	Max_length	Good's-coverage
IH1	24711235	59915	412.438204	262	435	0.999522
IH2	27128786	65139	416.475322	336	435	0.999426
IH3	26409833	63924	413.144249	336	523	0.999139
IH4	28180899	68967	408.61425	283	481	0.998756
IH5	25236680	61692	409.075407	229	432	0.999713
IH6	21362801	51384	415.748112	214	432	0.999617
IH7	28448957	68988	412.375442	228	444	0.998501
IH8	24397020	59354	411.042558	337	444	0.999649
IH9	27893090	68157	409.247619	228	433	0.999554
IH10	29816692	72450	411.548544	231	432	0.999043
IL1	25065981	60513	414.224729	237	469	0.999458
IL2	26222833	63424	413.452841	228	433	0.999426
IL3	30417780	73516	413.757277	236	474	0.998469
IL4	28024276	67238	416.792231	280	444	0.998756
IL5	26468399	64697	409.113236	259	436	0.999458
IL6	40079588	96184	416.697039	219	511	0.994866
IL7	30828777	73972	416.762789	261	540	0.998278
IL8	27279966	66294	411.499774	312	437	0.99882
IL9	25922417	62111	417.356298	288	432	0.999075
IL10	23993541	57563	416.822282	328	432	1
Summation		1325482				
Average	27394477.55	66274.1	413.3094102	266.6	454.95	0.9989763

Supplemental Table S3. Ruminal and intestinal microbial community alpha diversity indices

Item	Treatment		SEM	*P*-value
	L	H		
Rumen				
Community richness				
ACE	567.48	521.03	66.51	0.63
Chao	558.62	513.51	64.68	0.63
Sobs	550.10	509.60	63.66	0.66
Community diversity				
Shannon	4.17	3.92	0.15	0.26
Simpson	0.05	0.06	0.01	0.55
Intestinal				
Community richness				
ACE	503.82	403.93	67.16	0.31
Chao	494.25	398.33	64.75	0.31
Sobs	480.30	393.90	61.79	0.34
Community diversity				
Shannon	4.27	4.17	0.20	0.73
Simpson	0.04	0.05	0.01	0.81

L: low heat stress response; H: high heat stress response.

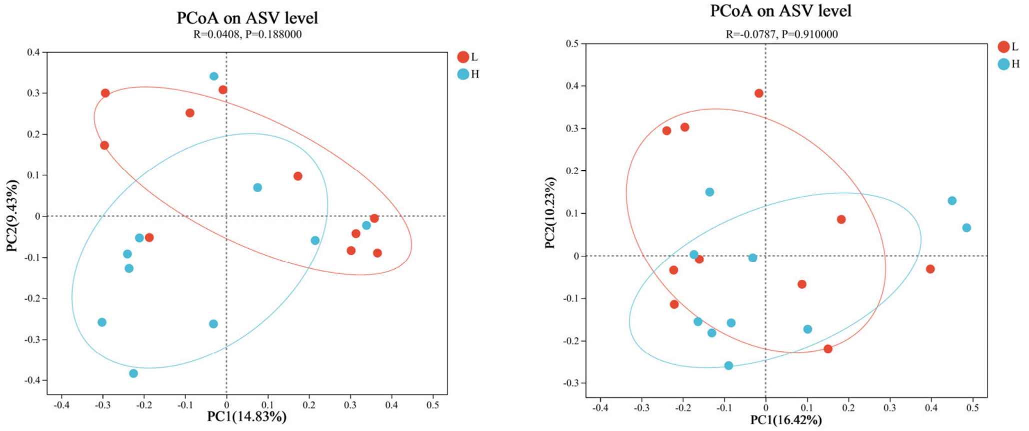

A B

Supplemental Figure S1. Beta diversity evaluated using the bray_curtis followed by ANOSIM and visualized using PCoA. A: rumen; B: intestinal. n = 10. L: low heat stress response, H: high heat stress response.

Supplemental Table S4. The order and sum abundance of the node of rumen microbiota in the intra-group symbiotic relationship analysis using co-occurrence network analysis as calves of different low heat stress response (L) or high heat stress response (H).

L			H		
Nodes	Sum abundance	Order	Nodes	Sum abundance	Order
g__Fusobacterium	0.0021739	1	*g__Eubacterium_nodatum_group*	0.0207638	1
g__U29-B03	0.0028085	2	*g__norank_o__RF39*	0.074762	2
g__Sphaerochaeta	0.0186403	3	*g__Prevotellaceae_UCG-001*	0.1223925	3
g__Synergistes	0.076376	4	*g__norank_f__Eubacterium_coprostanoligenes_group*	0.12948	4
g__Shuttleworthia	0.1103972	5	*g__Shuttleworthia*	0.1388505	5
g__Prevotellaceae_UCG-001	0.1241509	6	*g__Candidatus_Saccharimonas*	0.150084	6
g__norank_o__RF39	0.129358	7	*g__UCG-005*	0.2019835	7
g__Succiniclasticum	0.153421	8	*g__U29-B03*	0.2038866	8
g__norank_f__F082	0.1757476	9	*g__Alloprevotella*	0.2069016	9
g__Eubacterium_nodatum_group	0.1935885	10	*g__Sphaerochaeta*	0.2253546	10
g__Christensenellaceae_R-7_group	0.2864478	11	*g__norank_f__F082*	0.297084	11
g__UCG-005	0.3096494	12	*g__NK4A214_group*	0.2996533	12
g__Rikenellaceae_RC9_gut_group	0.3209787	13	*g__Lachnospiraceae_NK3A20_group*	0.3788386	13
g__Candidatus_Saccharimonas	0.337623	14	*g__UCG-002*	0.4261306	14
g__UCG-002	0.406752	15	*g__Rikenellaceae_RC9_gut_group*	0.454407	15
g__NK4A214_group	0.508552	16	*g__Christensenellaceae_R-7_group*	0.4818561	16
g__norank_o__Clostridia_UCG-014	0.74424	17	*g__Synergistes*	0.4850904	17
g__Ruminococcus	0.80265	18	*g__norank_f__Muribaculaceae*	0.536209	18
g__Lachnospiraceae_NK3A20_group	0.851247	19	*g__Ruminococcus*	0.5502332	19
g__norank_f__Muribaculaceae	0.965597	20	*g__norank_o__Clostridia_UCG-014*	0.7317288	20
g__Prevotella	1.58763	21	*g__Prevotella*	0.7602557	21

L: low heat stress response; H: high heat stress response.

Supplemental Table S5. The order and sum abundance of the node of intestinal microbiota in the intra-group symbiotic relationship analysis using co-occurrence network analysis as calves of different low heat stress response (L) or high heat stress response (H).

L			H		
Nodes	Sum abundance	Order	Nodes	Sum abundance	Order
g__Ruminococcus_torques_group	0.0509644	1	*g__Ruminococcus*	0.1041718	1
g__Subdoligranulum	0.0950672	2	*g__Parabacteroides*	0.1118909	2
g__Holdemanella	0.1163789	3	*g__Holdemanella*	0.15563	3
g__Ruminococcus	0.12746	4	*g__Bacillus*	0.1581298	4
g__Odoribacter	0.1492873	5	*g__norank_f__Eubacterium_coprostanoligenes_group*	0.1582229	5
g__Romboutsia	0.1516987	6	*g__Prevotella*	0.1607144	6
g__Alloprevotella	0.1519534	7	*g__Ruminococcus_torques_group*	0.1730172	7
g__Collinsella	0.164039	8	*g__Romboutsia*	0.1867661	8
g__Parabacteroides	0.17922	9	*g__Odoribacter*	0.197503	9
g__Bacillus	0.1984423	10	*g__Rikenellaceae_RC9_gut_group*	0.203136	10
g__Prevotella	0.2209358	11	*g__Collinsella*	0.21851	11
g__Bacteroides	0.287567	12	*g__norank_f__Erysipelotrichaceae*	0.2992047	12
g__Peptoclostridium	0.2930059	13	*g__Lactobacillus*	0.3470162	13
g__Faecalibacterium	0.338502	14	*g__Alloprevotella*	0.400147	14
g__UCG-005	0.560287	15	*g__Bacteroides*	0.4296844	15
g__norank_f__Erysipelotrichaceae	0.580603	16	*g__UCG-005*	0.5315638	16
g__Rikenellaceae_RC9_gut_group	0.6514647	17	*g__norank_f__Muribaculaceae*	0.783182	17
g__Lactobacillus	0.712985	18	*g__Peptoclostridium*	1.109116	18
g__Blautia	0.889286	19	*g__norank_o__Clostridia_UCG-014*	1.3546489	19
g__norank_f__Muribaculaceae	1.0706474	20			

L: low heat stress response; H: high heat stress response.

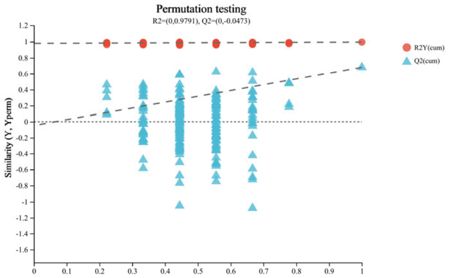

A

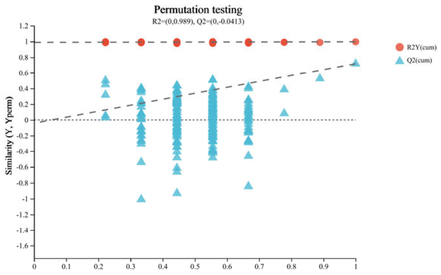

B

Supplemental Figure S2. Permutation validation plots comparing H and L groups in positive (A) and negative (B) ion mode. n = 10. L: low heat stress response, H: high heat stress response.

Supplemental Table S6. Differential metabolites and their corresponding enriched metabolic pathways identified in the serum metabolome of the host

Metabolite	ID	VIP_PLS-DA	FC(L/H)	*P*-value	RT	M/Z	KEGG Pathway Description
N-Methyl-2,4-Dihydroxy-3-Phenylquinoline	pos_1484	3.15	0.77	0.05	4.07	269.13	-
Val Ile	pos_1232	2.98	0.88	0.01	3.40	231.17	-
Propoxyphenyl Homohydroxysildenafil	pos_8211	2.31	0.95	7.422E-5	1.62	519.24	-
Gln Trp Trp	pos_8394	2.21	0.95	0.00	0.79	519.24	-
Lycoperodine I	pos_1754	2.10	0.94	0.02	4.76	217.10	-
1,2,3,4-Tetrahydro-3-Isoquinolinecarboxylic Acid	pos_7570	2.08	0.94	0.02	4.51	178.09	-
Pro Asn Ser	pos_1242	1.80	0.96	0.01	3.45	317.15	-
Gly Pro	pos_8203	1.67	0.94	0.02	1.66	227.10	-
Lys Met His	pos_3166	1.44	1.03	0.05	6.16	432.24	-
Pc(15:0/0:0)	pos_4050	1.49	0.97	0.01	6.81	504.30	-
Triphenylphosphine Oxide	pos_6103	1.32	1.02	0.02	6.12	279.09	-
Ile Ile	pos_7738	1.63	0.96	0.02	4.00	245.19	-
Dl-4-Hydroxyphenyllactic Acid	pos_8116	1.69	0.95	0.05	2.84	165.05	-
Acetyl-L-Cysteine	pos_8445	1.23	1.03	0.04	0.78	146.03	-
Ala Leu	pos_1678	1.29	0.97	0.02	4.65	243.13	-
Pyroglutamylisoleucine	neg_2288	1.27	0.97	0.02	4.65	241.12	-
Myristoyllysophosphatidylcholine	pos_5357	1.22	0.98	0.03	6.64	490.29	-
Thr Lys Lys	pos_3133	1.46	1.02	0.02	6.14	393.28	-
Alpha-Cyano-3-Hydroxycinnamic Acid	neg_7678	1.26	0.96	0.04	4.00	188.04	-
Pc(16:1/0:0)	pos_5355	1.14	0.98	0.03	6.64	516.30	-
Oleoyl-L-Alpha-Lysophosphatidic Acid	neg_5237	1.04	0.98	0.02	7.36	457.24	-
Mono(2-Ethylhexyl) Phthalate	neg_6022	0.87	1.01	0.00	6.26	277.14	-
Hexamethoxymethyl Melamine	pos_6314	0.84	1.01	0.01	6.00	391.23	-

Ethylmorphine	pos_6339	2.90	1.19	0.01	5.98	331.20	-
Arecoline	pos_8296	2.42	0.87	0.03	1.55	173.13	-
4-Heptylphenol	pos_3082	1.47	1.03	0.01	6.12	193.16	-
2-Methylhippuric Acid	neg_1552	2.00	0.94	0.02	2.84	192.07	-
3-Hydroxybenzoic Acid	neg_6616	1.27	1.04	0.05	5.97	137.02	Phenylalanine, tyrosine and tryptophan biosynthesis
Dibutyl Phthalate	pos_5849	1.01	1.01	0.00	6.28	279.16	-
Diethyl Phthalate	pos_3381	0.95	1.01	0.00	6.28	205.09	-
Ur-12670	pos_7765	0.98	0.98	0.04	3.94	456.28	-
Norbutorphanol	neg_5422	2.16	0.93	0.01	6.79	517.30	-
Actarit	pos_1088	1.99	0.94	0.03	2.84	194.08	-
2,6-Dimethylaniline	pos_5524	0.79	1.01	0.00	6.47	122.10	Drug metabolism - cytochrome P450
Ponasterone A	pos_5706	0.99	1.01	0.04	6.33	528.33	-
Tridecanedioic Acid	neg_6228	0.93	1.02	0.02	6.15	243.16	-
Elaidic Acid	pos_907	1.02	1.01	0.00	1.53	283.26	-
Geranyl Acetoacetate	neg_6180	0.86	1.01	0.02	6.18	237.15	-
(R)-2-Hydroxysterculic Acid	pos_5652	1.09	1.02	0.01	6.37	328.28	-
(R)-1-Octen-3-Ol	neg_3482	0.95	1.01	0.01	6.16	173.12	-
Oleamide	pos_3745	0.84	1.01	0.04	6.53	282.28	-
Pc(22:5/0:0)	pos_5452	1.32	0.98	0.01	6.54	592.34	Glycerophospholipid metabolism;Choline metabolism in cancer
Lysope(0:0/22:4(7Z,10Z,13Z,16Z))	pos_4062	1.50	0.96	0.03	6.82	530.32	-
Pe(14:1(9Z)/14:0)	pos_3456	0.87	1.01	0.01	6.30	634.45	Metabolic pathways;Glycerophospholipid

							metabolism;Glycosylphosphatidylinositol (GPI)-anchor biosynthesis;Autophagy - other;Kaposi sarcoma-associated herpesvirus infection;Pathogenic Escherichia coli infection;Retrograde endocannabinoid signaling;Autophagy - animal
10-Nitrolinoleic Acid	neg_3336	1.52	0.97	0.02	6.09	384.24	-
Glyceryl Palmitate	pos_8305	0.80	1.01	0.01	1.49	331.28	-
(S)-(+)-Curcumene	pos_6131	1.30	1.03	0.03	6.11	175.15	-
Methylprednisolone Acetate	pos_3167	1.51	1.04	0.04	6.16	458.25	-
Capsicoside A1	pos_7479	1.00	0.98	0.04	4.71	636.41	-
Cholestane-3,7,12,25-Tetrol-3-Glucuronide	pos_1688	0.97	0.99	0.04	4.66	676.41	Metabolic pathways; Pentose and glucuronate interconversions; Bile secretion; Ascorbate and aldarate metabolism; Biosynthesis of cofactors
Spinacoside D	neg_2368	2.39	0.90	0.01	4.79	799.36	-
(1R*,2R*,4R*,8S*)-P-Menthane-1,2,8,9-Tetrol 9-Glucoside	neg_7338	1.67	0.96	0.01	4.79	777.38	-
Alisol A	pos_6575	1.15	1.02	0.00	5.88	455.35	-
(3Beta,17Alpha,23R)-17,23-Epoxy-3,29-Dihydroxy-27-Norlanost-8-Ene-15,24-Dione	neg_5588	0.98	1.02	0.02	6.55	471.31	-
Cdp-Dg(I-16:0/20:5(6E,8Z,11Z,14Z,17Z)-O	neg_2624	1.91	0.94	0.03	5.27	1036.46	-

h(5))							
Pc(2:0/20:5(7Z,9Z,11E,13E,17Z)-3Oh(5,6,15))	neg_7909	1.91	0.95	0.01	3.49	630.31	-
Pa(Lte4/2:0)	neg_7081	2.10	0.96	0.00	5.30	616.24	-
Pe(20:5(7Z,9Z,11E,13E,17Z)-3Oh(5,6,15)/P-18:1(9Z))	pos_4936	1.74	0.97	0.00	7.32	800.48	-
Pi(5-Iso Pgf2Vi/20:0)	pos_4958	1.47	0.97	0.02	7.31	978.60	-
Xanthosine	neg_8367	1.52	0.95	0.02	1.58	283.07	Metabolic pathways; ABC transporters; Nucleotide metabolism; Purine metabolism; Caffeine metabolism
L-Thyronine	pos_1228	2.99	0.85	0.02	3.38	315.13	-
3-[4-(3-Trifluoromethyl-Phenyl)-Piperazin-1-Yl]-Dihydrofuran-2-One	neg_8001	2.13	0.91	0.02	3.37	313.12	-
Tryptophylhydroxyproline	pos_6804	2.42	0.89	0.04	5.61	322.12	-
Glutamyltryptophan	pos_2047	1.99	0.91	0.04	5.18	316.13	-
Hydroxyprolyl-Gamma-Glutamate	neg_1366	2.32	0.95	6.208E-5	1.62	517.23	-
4-Alanylaminoantipyrine	neg_7192	2.33	0.88	0.04	5.04	309.11	-
Ceanothine E	pos_7949	1.88	0.95	0.01	3.49	632.32	-
Angiotensin Iv	pos_7427	1.83	0.96	0.01	4.79	779.39	Renin-angiotensin system
Glycylvaline	neg_8329	2.03	0.94	0.03	1.65	155.08	-
N-Alpha-Acetyl-L-Lysine	neg_7897	1.98	0.94	0.04	3.52	169.10	-
Asparaginyl-Phenylalanine	pos_7446	1.97	0.95	0.03	4.76	244.11	-
Formyllysine	pos_8204	1.96	0.95	0.04	1.65	157.10	-
N-(3-Amino-3-Oxopropyl)-L-Valine	pos_7943	1.88	0.95	0.05	3.53	171.11	-
N-Methyl-L-Proline	pos_997	1.81	0.96	0.04	1.65	130.09	-

Beta-Hydroxy-Gamma-Trimethylaminobutyric Acid	pos_1259	1.82	0.96	0.05	3.53	144.10	-
2,6-Diaminopimelic Acid	pos_877	1.73	0.97	0.03	1.17	155.08	Metabolic pathways;D-Amino acid metabolism;Lysine biosynthesis;Biosynthesis of amino acids
1-Pyrroline-5-Carboxylic Acid	pos_8377	1.73	0.96	0.03	0.84	155.08	Metabolic pathways;Alanine, aspartate and glutamate metabolism;Arginine and proline metabolism;Biosynthesis of amino acids
L-Felinine	pos_1469	1.50	1.04	0.01	4.04	190.09	-
N-Acetyl-L-Tyrosine	neg_7841	1.77	0.95	0.05	3.63	222.08	-
Allysine	pos_8329	1.61	0.97	0.03	1.16	128.07	Metabolic pathways;Lysine degradation;Lysine biosynthesis;Biosynthesis of amino acids
Libenzapril	pos_954	1.48	0.97	0.04	1.56	418.19	-
N-Acetyl-D-Cysteine	neg_8394	1.42	1.06	0.01	1.56	162.02	-
Carfilzomib	pos_7440	1.29	0.98	0.01	4.76	720.43	-
Glu-Thr	neg_944	1.31	1.03	0.05	0.72	247.09	-
2-N-Propylthiazolidine-4-Carboxylic Acid	pos_8052	1.25	1.03	0.03	3.16	176.07	-
Gamma-Glutamylthreonine	pos_687	1.44	1.03	0.04	0.72	249.11	-
N-Acetyl-Dl-Valine	neg_1857	1.13	0.98	0.03	3.58	158.08	-
Gluten Exorphin C	pos_1646	0.94	0.99	0.05	4.56	632.38	-
4-Allylpyrocatechol Sulfate	neg_4164	2.97	1.21	0.01	6.64	229.02	-

Malic Acid	neg_988	1.12	0.98	0.01	0.76	133.01	Metabolic pathways;Glyoxylate and dicarboxylate metabolism;Renal cell carcinoma;Pathways in cancer;Proximal tubule bicarbonate reclamation;Carbon metabolism;Pyruvate metabolism;Glucagon signaling pathway;Citrate cycle (TCA cycle);Central carbon metabolism in cancer;Taste transduction
Fumaric Acid	neg_8717	1.23	0.98	0.01	0.76	115.00	Metabolic pathways;Arginine biosynthesis;Renal cell carcinoma;Pathways in cancer;Carbon metabolism;Pyruvate metabolism;Nicotinate and nicotinamide metabolism;Alanine, aspartate and glutamate metabolism;Glucagon signaling pathway;Phenylalanine metabolism;Tyrosine metabolism;Citrate cycle (TCA cycle);Butanoate metabolism;Central carbon metabolism in cancer;Oxidative phosphorylation
10-Hydroxydecanoic Acid	neg_3588	1.31	1.03	0.03	6.20	187.13	-

(S)-5-Amino-3-Oxohexanoate	pos_827	1.63	0.96	0.03	0.84	128.07	Metabolic pathways;Lysine degradation
2-Oxo-5-(3,3-Dimethylguanidine-1-Yl)Pentanoic Acid	pos_770	2.28	1.07	0.00	0.78	202.12	-
Acetone Cyanohydrin	neg_7977	1.93	0.95	0.05	3.40	169.10	Metabolic pathways
Neamine	pos_8493	1.77	1.05	0.01	0.75	345.18	Neomycin, kanamycin and gentamicin biosynthesis
Isomaltose	pos_8596	1.64	1.04	0.01	0.63	325.11	Metabolic pathways;Starch and sucrose metabolism
5-Methylthioribose	neg_4315	1.22	1.02	0.00	6.86	239.06	Metabolic pathways;Cysteine and methionine metabolism
Anhydroerythromycin	pos_1786	1.30	0.98	0.01	4.81	680.44	-
2-(Acetylamino)-2-Deoxy-Alpha-D-Mannopyranose	pos_8557	1.09	1.02	0.04	0.67	204.09	-
Fructose	neg_6758	1.25	0.98	0.01	5.86	201.04	Amino sugar and nucleotide sugar metabolism;Biosynthesis of nucleotide sugars
Metyrapone	neg_6515	2.06	0.90	0.05	6.02	271.11	-
2-Amino-4-Methylpyridine	pos_8378	1.75	0.95	0.03	0.84	109.08	-
Deethylatrazine	pos_2191	2.84	0.83	0.04	5.34	205.10	-
Phthalic Anhydride	pos_3365	1.00	1.01	0.00	6.28	149.02	-
11-Beta-Hydroxyandrosterone-3-Glucuronide	neg_8618	2.45	0.93	0.00	0.78	517.23	Metabolic pathways;Biosynthesis of cofactors;Pentose and glucuronate interconversions;Bile secretion;Ascorbate and aldarate metabolism;Tryptophan metabolism

Imetit	pos_6468	1.77	0.95	0.04	5.90	193.05	-
Indole	pos_1628	2.23	0.92	0.02	4.51	159.09	Metabolic pathways;Phenylalanine, tyrosine and tryptophan biosynthesis;Tryptophan metabolism;Protein digestion and absorption
Indoleacrylic Acid	pos_1753	1.84	0.95	0.03	4.76	188.07	-
Indole-3-Carboxaldehyde	pos_6733	1.10	0.98	0.04	5.71	146.06	-
3H-Indole-3-Propanoic Acid,A-Amino-	pos_1629	2.15	0.94	0.01	4.51	205.10	-
L-4-Chlorotryptophan	pos_6466	1.72	0.96	0.04	5.90	239.06	-
3H-2,3-Benzodiazepine	neg_2562	2.25	0.89	0.02	5.13	203.08	-
Nicotyrine	neg_7469	2.10	0.94	0.01	4.51	203.08	-
5-(3-Pyridyl)-2-Hydroxytetrahydrofuran	pos_1808	1.66	0.95	0.01	4.85	166.09	Chemical carcinogenesis - DNA adducts;Metabolism of xenobiotics by cytochrome P450
Alpha-Bisabolol Oxide B	pos_5985	1.35	1.03	0.03	6.19	256.23	-
2,2,6,6-Tetramethyl-4-Piperidinol	pos_6046	1.03	1.01	0.00	6.16	158.15	-
2-(Methylthio)Benzothiazole	pos_5953	0.87	1.01	0.00	6.21	182.01	-
Bisbenzimide	neg_7163	1.41	0.97	0.02	5.11	473.20	-
Abt-510	pos_4319	1.84	0.96	0.00	7.33	1026.66	-
Theophylline	neg_748	0.97	1.01	0.00	0.61	215.03	Metabolic pathways;Caffeine metabolism
3-Propyl-1H-Pyrazole	pos_8205	1.96	0.94	0.04	1.65	111.09	-
Fluvastatin	neg_5409	1.12	1.02	0.04	6.80	470.20	Bile secretion
5-Methoxytryptophan	neg_2341	1.92	0.94	0.03	4.76	215.08	-
Trisjuglone	neg_7940	1.02	1.02	0.02	3.43	515.04	-

3-(2-Hydroxyphenyl)Propanoic Acid	neg_2830	2.29	1.14	0.02	5.71	165.06	Metabolic pathways;Phenylalanine metabolism
Alpha-Methyl-M-Tyrosine	neg_2240	2.09	0.94	0.01	4.51	176.07	-
Urolithin B	pos_6467	1.76	0.96	0.03	5.90	212.05	-

Supplementary Table S7. Properties of networks.

Items	Degree	Closeness centrality	Betweenness centrality
Rumen			
Phenotype			
Respiration rate	6	11.94	400
Rectal temperature	2	8.61	52
Bacteria			
Ruminococcus	5	11.44	261
Clostridium_sensu_stricto_1	5	10.18	262
Allorhizobium-Neorhizobium-Pararhizobium-Rhizobium	5	10.90	245
Jeotgalibaca	3	8.16	144
Intestinimonas	3	10.44	104
norank_f__Weeksellaceae	2	8.68	280
Prevotella	1	5.50	0
Pseudoramibacter	1	7.79	0
Metabolites			
(S)-5-Amino-3-Oxohexanoate	4	10.43	84
2,6-Diaminopimelic Acid	4	10.43	84
Allysine	4	10.43	84
Fumaric Acid	3	9.90	96
Malic Acid	3	9.53	304
Serum			
HSP-70	4	9.07	262
INS	3	7.35	102
IL-4	2	8.18	184
TFA	2	7.33	0
GLU	2	7.26	144
IgA	2	6.58	100
IL-6	2	5.86	52
Cortisol	1	7.51	0
IL-2	1	7.05	0
TNF-α	1	6.51	0
T3	1	6.49	0
HP	1	5.50	0
SAA	1	4.72	0
Intestinal			
Phenotype			
Respiration rate	7	11.70	102.67
Rectal temperature	6	11.78	63.33
Bacteria			
Pseudoramibacter	8	13.12	147.93
Catenisphaera	5	11.25	167.87

norank_f__Oscillospiraceae	5	11.45	39.07
Colidextribacter	4	9.70	24.20
Olsenella	3	9.20	12.80
Family_XIII_AD3011_group	3	9.70	0.00
Metabolites			
(S)-5-Amino-3-Oxohexanoate	3	8.38	0.00
2,6-Diaminopimelic Acid	3	8.38	0.00
Allysine	3	8.38	0.00
Malic Acid	1	1.00	0.00
Fumaric Acid	1	1.00	0.00
Serum			
HSP-70	3	9.37	44.13
IL-6	3	9.08	134.00
TFA	2	8.28	12.00
IgA	2	7.33	72.00
IL-2	2	7.13	0.00
GLU	2	6.20	38.00
TNF-α	1	7.53	0.00
SAA	1	6.42	0.00
T3	1	6.54	0.00
IL-4	1	4.85	0.00
INS	1	1.00	0.00
HP	1	1.00	0.00

Supplementary Table S8. Prediction of serum metabolites and bacteria in response to host heat stress using area under the receiver operating characteristic curve (AUC).

Items	AUC	Confidence interval		*P* value
Serum				
HSP-70	0.800	0.597	1.000	0.023
IL-2	0.800	0.585	1.000	0.023
INS	0.765	0.543	0.988	0.045
TFA	0.760	0.534	0.987	0.049
HP	0.640	0.378	0.902	0.290
IgA	0.650	0.400	0.900	0.257
TNF-α	0.635	0.377	0.893	0.308
T3	0.640	0.389	0.891	0.290
Cortisol	0.630	0.378	0.882	0.326
SAA	0.590	0.332	0.848	0.496
GLU	0.575	0.313	0.837	0.571
IL-4	0.550	0.275	0.825	0.706
IL-6	0.540	0.278	0.802	0.762
Metabolites				
Malic Acid	0.870	0.711	1.000	0.005
Fumaric Acid	0.860	0.698	1.000	0.007
Allysine	0.780	0.566	0.994	0.034
(S)-5-Amino-3-Oxohexanoate	0.780	0.569	0.991	0.034
2,6-Diaminopimelic Acid	0.770	0.550	0.990	0.041
Rumen Bacteria				
g__Ruminococcus	0.780	0.551	1.000	0.034
g__Intestinimonas	0.790	0.584	0.996	0.028
g__Pseudoramibacter	0.760	0.535	0.986	0.049
g__Allorhizobium-Neorhizobium-Pararhizobium-Rhizobium	0.760	0.539	0.981	0.049
g__Prevotella	0.760	0.539	0.981	0.049
g__Clostridium_sensu_stricto_1	0.750	0.526	0.975	0.059
g__norank_f__Weeksellaceae	0.700	0.462	0.938	0.131
g__Jeotgalibaca	0.700	0.462	0.938	0.131
Intestinal Bacteria				
g__Pseudoramibacter	0.850	0.665	1.000	0.008
g__norank_f__Oscillospiraceae	0.820	0.636	1.000	0.016
g__Colidextribacter	0.780	0.557	1.000	0.034
g__Olsenella	0.780	0.566	0.994	0.034
g__Family_XIII_AD3011_group	0.780	0.563	0.997	0.034
g__Catenisphaera	0.700	0.462	0.938	0.131
